# Analysis of the Results of Severe Intraepithelial Squamous Cell Lesions and Preinvasive Cervical Cancer Phototheranostics in Women of Reproductive Age

**DOI:** 10.3390/biomedicines10102521

**Published:** 2022-10-09

**Authors:** Aida Gilyadova, Anton Ishchenko, Anatoly Ishenko, Svetlana Samoilova, Artem Shiryaev, Alevtina Kiseleva, Natalya Petukhova, Kanamat Efendiev, Polina Alekseeva, Evgeny Stranadko, Victor Loschenov, Igor Reshetov

**Affiliations:** 1 University Clinical Hospital No. 1, Levshin Institute of Cluster Oncology, Sechenov First Moscow State Medical University (Sechenov University), Ministry of Health of the Russian Federation, 119435 Moscow, Russia; 2 National Medical Research Treatment and Rehabilitation Center, Ministry of Health of the Russian Federation, 125367 Moscow, Russia; 3 Prokhorov General Physics Institute of the Russian Academy of Sciences, 119991 Moscow, Russia; 4 Department of Laser Micro-, Nano-, and Biotechnology, Institute of Engineering Physics for Biomedicine, National Research Nuclear University “MEPhI”, 115409 Moscow, Russia; 5 Skobelkin Scientific and Practical Center for Laser Medicine FMBA, 121165 Moscow, Russia

**Keywords:** human papillomavirus, photodynamic therapy, intraepithelial squamous lesions of the cervix, preinvasive cervical cancer, cervical cancer, fluorescence diagnostics, photosensitizers, cervical intraepithelial neoplasia, HPV, colposcopy, conization, ablation, PDT photosensitizers, phototheranostics

## Abstract

(1) Purpose: To investigate the efficacy and safety of using PDT in the treatment of severe intraepithelial squamous lesions of the cervix and preinvasive cervical cancer associated with HPV in women of reproductive age. (2) Methods: The examination and treatment of 45 patients aged 22–49 years with morphologically confirmed HPV-associated cervical intraepithelial neoplasia of a severe degree (17 patients) and preinvasive cervical cancer (28 patients) were performed. All patients underwent PDT of the cervix using a chlorin e6 photosensitizer; after which, the affected areas of the cervix were evaluated using video and spectral fluorescence diagnostics. PDT effectiveness was assessed on the basis of colposcopy data, a cytological examination of exo- and endocervix and PAP test scrapings or the liquid cytology method, and polymerase chain reaction for HPV carriage 4 weeks after PDT, as well as on the basis of histological and immunohistochemical studies of biopsy materials 5 weeks after PDT. The expression levels of the Ki-67 and p16 markers in the affected areas of the cervix were also assessed. (3) Results. All patients included in the study tolerated the intravenous administration of the photosensitizer well, with no side effects or allergic reactions observed. In 88.2% of patients with CIN III/HSIL and in 85.7% of women with preinvasive cervical cancer, the effect of the treatment was noted after the first PDT procedure, while complete regression of the dysplasia foci was observed in 15 women (88.2%) with CIN III/HSIL and in 25 patients (89.3%) with preinvasive cervical cancer. Partial regression to the form of LSIL/CIN I was noted in two cases (11.8%) in the CIN III/HSIL group and in three cases (10.7%) in the group of patients with preinvasive cervical cancer. After PDT, a statistically significant decrease in the expression of the Ki-67 and p16 levels relative to the initial values was noted. (4) Conclusions. The results obtained indicate the high efficiency of PDT with intravenous administration of the chlorin photosensitizer for the treatment of intraepithelial lesions of the cervix with a selective effect on pathologically altered tissue. The use of this approach makes it possible to preserve the normal anatomical and functional characteristics of the cervix, which is especially important for maintaining the fertility of patients.

## 1. Introduction

Cervical cancer (CC) remains one of the most common oncological diseases of the female reproductive system [1,2]. Cervical intraepithelial neoplasia (CIN), which is considered a precancerous lesion of the cervical epithelium, is caused by persistent human papillomavirus infection with high carcinogenic risk (HPV-HCR) [2,3,4]. To date, it has been established that the risk of CC in women with CIN is 20 times higher than in healthy women [3,4,5].

According to the 2014 WHO classification, CIN lesions are classified as low-grade squamous intraepithelial lesions (LSIL) and high-grade squamous intraepithelial lesions (HSIL) [5]. Most clinically used treatments for SIL lesions and cervical HPV infection, such as diathermocoagulation, cryotherapy, laser vaporization, and laser or electrosurgical excision, are invasive. Their use can cause such complications as bleeding, endometriosis, and cervical stenosis [3,6]. In subsequent pregnancies, the adverse effects of using these methods during treatment may manifest as spontaneous miscarriage, premature birth, and low and extremely low birth weights of newborns [6,7].

Since HPV infection is often diagnosed in women of childbearing age with a high probability of reinfection after treatment, it seems necessary to search for and test the application of alternative treatment methods for precancerous cervical changes that would be no less effective than traditional approaches but with a lower incidence of side effects [3,8,9,10,11,12,13].

It was shown that the selective effect of PDT in areas of photosensitizer accumulation triggers the destruction of tumor tissue. At the same time, PS has a minimal effect on nearby areas of healthy tissues due to its more pronounced accumulation in tumor tissues compared to healthy ones. Nowadays, the method has proven itself in the treatment of a number of skin and mucous membranes diseases, the uses of which, in these cases, are characterized by high clinical efficacy and safety [13,14]. The advantages of this method include rapid healing of the affected area, minimal scarring, and the possibility of treating multiple lesions simultaneously. It is important to note that PDT selectively affects tumor cells without damaging neighboring healthy cells [14,15,16,17,18].

The use of PDT has shown effectiveness in the treatment of infectious diseases caused by HPV-HCR, including genital warts, vaginal intraepithelial neoplasia, or vulvar intraepithelial neoplasia [17,18,19]. However, there are only a few studies on the use of this method in the treatment of precancerous lesions and CC.

Active screening among the female population for precancerous lesions and their treatment, if detected, especially in developing countries, provides very high chances of reducing mortality from CC. Given the rather high prevalence of CC in young patients of reproductive age, this determines the importance of maintaining fertility when using this method of treatment. At the same time, reports on the use of the proposed method of treatment for severe intraepithelial squamous lesions of CIN and carcinoma in situ (CIS) are isolated and unorganized.

All this indicates the need for an in-depth analysis of the results of the PDT use in precancerous lesions of the cervix and CC in order to improve the clinical efficacy and safety of this treatment.

The term “phototheranostics” refers to the combined use of spectral and video fluorescence diagnostics methods and treatment using PDT. This is an effective, relatively safe, minimally invasive treatment and diagnostic method for precancerous changes in the cervical epithelium, the use of which makes it possible to achieve the eradication of HPV infection as well.

According to the results of a previous study on the effectiveness of intraepithelial cervical lesions and neoplasia treatment, including preinvasive, microinvasive, and squamous CC associated with HPV infection, based on the use of the phototheranostics method, a high clinical efficacy and safety of this approach was demonstrated [20]. Considering these data, it seems appropriate to conduct further research on the in-depth study of the possibilities of using phototheranostics in the forecited pathology.

The aim of the study was to investigate the efficacy and safety of using the method of photodynamic therapy in the treatment of severe intraepithelial squamous lesions of the cervix and CIS associated with HPV infection in women of reproductive age.

## 2. Materials and Methods

### 2.1. Patients

The study was conducted at I.M. Sechenov First Moscow State Medical University Clinical Hospital No. 1. It included 45 patients aged 22–49 years with morphologically confirmed HPV-associated severe intraepithelial neoplasia of the cervix and CIS. The protocol was approved by the local ethics committee of the L.L. Levshin on the basis of the University Clinical Hospital No. 1 of Sechenov University. All patients signed informed consent to participate in the study.

The distribution of the patients according to the characteristics of their lesions is shown in Table 1. Of the 45 patients, 17 (27.8%) had severe intraepithelial squamous lesions, and 28 women (62.2%) were diagnosed with CIS.

Intraepithelial lesions of the cervix were verified using histological and cytological methods determined before PDT.

Prior to PDT, the following oncogenic HPV types were identified in all patients using polymerase chain reaction (PCR): in 18 patients (40.0%)—type 16, in 14 women (31.1%)—type 18, in 6 patients (13.4%)—type 33, in 2 patients (4.4%)—type 11, in 3 patients (6.7%)—type 35, and in 2 patients (4.4%)—type 56 (Table 2).

### 2.2. Photosensitizer

Patients were administered a chlorin e6 photosensitizer 2 h before the procedure. In this study, the active substance of the photosensitizer is the imegluminum chlorinum e6 sodium salt of chlorin e6 (Ce6)—Fotoditazin ^®^ (Company “Veta-Grand”,Moscow, Russia, registration certificate number LS-001246 from 18.05.2012). The maximum accumulation contrast of Ce6 in the tumor tissue was observed 2–3 h after intravenous injection.

The calculated dose of Fotoditazin ^®^—1 mg/kg of body weight—was dissolved in 200 mL of 0.9% sodium chloride solution. Two hours before the start of the procedure, each patient was infused with a solution, prepared ex tempore, intravenously for 30 min, which was administered in a darkened room. The patients were instructed about the importance of observing a strict light regimen: avoiding exposure to direct sunlight, insolation, viewing gadgets, the need to reduce the brightness on the phone screen to a minimum, use of sunglasses, and UVA/UVB SPF sunscreens from the moment the solution with a photosensitizer was introduced until 48 h after the PDT procedure.

Of the possible side effects of the drugs, it should be noted: an increase in body temperature (37–38 °C) for 30 min, pain in the area of impact of laser irradiation, increased blood pressure in patients with concomitant diseases of the cardiovascular system, mild phototoxicity of the skin in the form of hyperemia, and allergic reactions. The half-life of the drug, according to the instructions, is 12 h; 28 h after intravenous administration, trace amounts of the drug were found in the blood.

### 2.3. Phototheranostics

#### 2.3.1. Fluorescence Diagnostics

**Figure 1:**
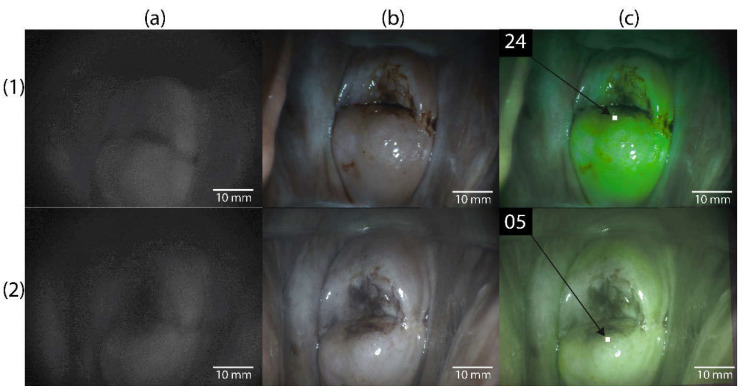
Images of cervical tissue from a patient with CIN III. (**1**) Before PDT. (**2**) After PDT. (**a**) Black and white mode. (**b**) Color mode. (**c**) Combined mode (in the upper left corner of the figures is the fluorescence index in rel. units).

PDT of the cervix was performed in the operating room under intravenous anesthesia. A targeted biopsy of the affected areas of the cervical mucosa was performed using video fluorescence imaging. A two-channel fluorescent video system was used to perform video fluorescence diagnostics [20]. The video system provides real-time color imaging over which a fluorescent image is superimposed with a fluorescence display in green (Figure 1 ), which allows determining the boundaries of neoplasms. While performing video- fluorescence diagnostics, the degree of accumulation of Ce6 in the cervical mucosa was quantitatively assessed by the contrast index [21].

Before the diagnostics, each patient underwent normalization of the contrast index in the area with the lowest fluorescent signal (normal cervical mucosa), which was assigned a contrast index value of 10 rel. units. Video fluorescence diagnostics at the beginning of the study made it possible to identify areas of the highest PS accumulation, which is typical for pathologically altered tissues (contrast index > 10 rel. units). 

After preliminary processing of the external genital organ’s skin, the vaginal mucosa, and the vaginal portion of the cervix under aseptic conditions, the cervix is exposed in the mirrors.

With the help of a fiber spectrometer, normal and pathologically altered cervical tissue (ectocervix and endocervix) were assessed with a quantitative measurement of the contrast indices at several points (2 points in each quadrant of the ectocervix circle and 1 point of the endocervix before the start of photodynamic therapy by the spectral fluorescence method).

**Figure 2:**
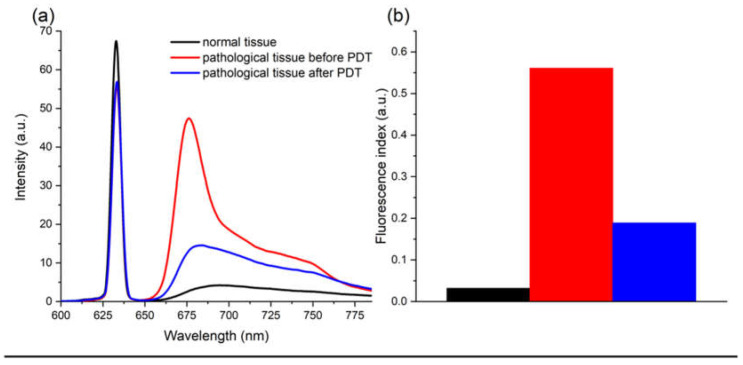
Results of the spectral diagnostics. (**a**) Fluorescence spectra of the cervical canal before and after PDT normalized to the laser line. (**b**) Fluorescence index chart.

For spectral fluorescence diagnostics, a fiber spectrometer with PS fluorescence excitation by a He-Ne laser (λ = 632.8 nm, Pmax = 15 mW) was used. Spectral fluorescence diagnostics was performed using a Y-shaped optical fiber. In the process of diagnosis, the fluorescence spectra of normal and pathologically altered cervix tissue were assessed (Figure 2). Registration was performed before and after PDT. In each case, the fluorescence index was calculated.

#### 2.3.2. Photodynamic Therapy

Phototheranostics was performed in several stages: first, a solution of a chlorin e6 photosensitizer was injected into the patient’s body; then, the affected areas of the cervix were assessed using video fluorescence and spectral fluorescence diagnostics. The studied areas were periodically washed of blood or sanies secretions. After that, the tumor tissue, which accumulated in the photosensitizer, was treated with light radiation with the appropriate wavelength directly with PDT.

PDT was performed using a semiconductor laser source with a generation wavelength of 660 ± 5 nm and an output power of up to 2 W. The doses of light energy during PDT of the mucous vaginal portion of the cervix amounted to 250–300 J/cm^2^ and, for the columnar epithelium of the cervical canal, 200 J/cm^2^. The average power density for impact on the cervical tissue was 0.29 W/cm^2^ and, for the cervical canal, 0.25 W/cm^2^. To irradiate the endocervix, an optical fiber with a cylindrical diffuser 15 mm long was used, which made it possible to carry out equable irradiation along the entire surface of the cervical canal. After polypositional therapy of the ectocervix with laser radiation with a spot diameter of 1–1.5 cm and the endocervix, contrast indices were quantitatively recorded at the same points of normal and pathologically altered cervix tissue (ectocervix and endocervix) by the spectral fluorescence method.

PS photobleaching was calculated by analyzing the fluorescence indices before and after PDT, which made it possible to evaluate the effectiveness of the therapy. In the case of insufficient photobleaching of PS, additional irradiation was performed. According to the results of a number of studies, photobleaching at the level of 50–70% is the most effective [22,23,24,25,26].

All procedures for the assessment and processing of fluorescence spectra were performed using the Uno Momento program developed in the Laboratory of Laser Biospectroscopy of the GPI RAS.

In all patients included in the study, images of the cervical mucosa were obtained before and after PDT in three modes: black and white (fluorescent), color, and combined.

### 2.4. Laboratory and Instrumental Research Methods

The effectiveness of phototheranostics in the treatment of intraepithelial neoplasia of the cervix was assessed based on the data of extended colposcopy, cytological examination of exo- and endocervix and PAP test scrapings or the liquid cytology method, and real-time PCR for HPV carriage 4 weeks after PDT, as well as according to the results of the histological verification and immunohistochemistry of the biopsy material 5 weeks after the end of the PDT course.

While conducting histological and immunohistochemical research methods, the expression levels of markers Ki-67 and p16 were evaluated. As material for the study, a biopsy sample of the cervical mucosa was used, which was fixed in 10% buffered pH-neutral formalin and subjected to the standard automated histological processing. Micro-preparations were stained with hematoxylin–eosin. With light microscopy, the degree of squamous epithelial lesion (degree of dysplasia) was assessed in accordance with the WHO classification of Tumors 5th edition Female Genital Tumor.

Immunohistochemical staining with antibodies of CINtec p16 (Histology, Ventana) and Ki-67 (30-9, Ventana) was performed on a BENCHMARK XT immunohistotainer (Ventana Medical Systems Oro Valley, Arizona, USA,). The expression of p16 was defined as positive, while a “blocky”, strong nuclear, and cytoplasmic expression in a continuous segment of cells (at least 10–20 cells), Ki-67 was defined as the percentage of nuclei with positive staining in the epithelial layer.

Statistical processing of the obtained data was carried out using the StatSoft STATISTICA 10 (Dell, Round Rock, Texas, USA) and Microsoft Excel 2016 software packages (One Microsoft Way, Redmond, Washington, USA ). The distribution normality was assessed using the Kolmogorov–Smirnov test. Continuous quantitative parameters were presented as the median and lower and upper quartiles: Me (Q25, Q75). Comparison of the indicators in related samples was carried out using the Wilcoxon test.

For all qualitative indicators, the frequency of occurrence of signs as a percentage of the number of groups was calculated. Frequency comparisons were performed using the chi-square test or Fisher’s exact test for pairwise comparisons. Differences were considered significant at *p* < 0.05.

## 3. Results

All patients included in the study tolerated intravenous PS administration. There were no side effects or allergic reactions. In six women, slight phototoxicity of the skin was noted within a day after the injection, which manifested in the form of hyperemia, but the symptoms quickly stopped without taking any measures within 2 days.

In 15 out of 17 patients with CIN III/HSIL (88.2%), the effect of the treatment was noted after the first PDT procedure and, in 2 women (11.8%), after the second therapy session (Table 3). In the group with CIS, in 24 patients (85.7%), the effect was detected after the first PDT procedure and, in four cases (14.4%), after the second session. In the entire sample of 39 women (86.7%), the effect was observed after the first session and, in 6 patients (13.3%), after the second treatment with PDT.

An analysis of the treatment results showed that complete regression of the dysplasia foci was observed in 15 women (88.2%) with severe intraepithelial squamous lesions and in 25 patients (89.3%) with CIS (Table 4). At the same time, partial regression in the form of LSIL/CIN I was noted in two cases (11.8%) in the CIN III/HSIL group and in three cases (10.7%) in the group of patients with CIS.

**Figure 3:**
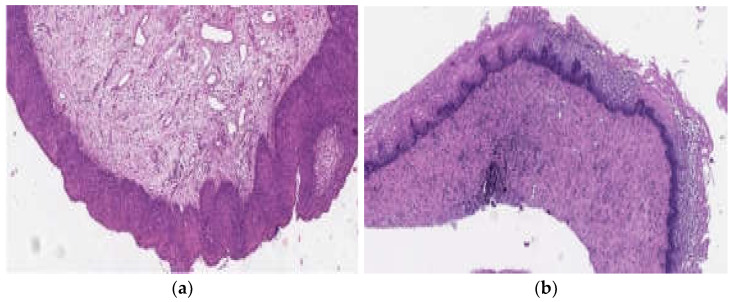
Histological preparations of a patient with HSIL\CIN III. Hematoxylin–eosin. Boost 90: (**a**) before phototheranostics and (**b**) 5 weeks after phototheranostics.

Figure 3 shows histological preparations of a patient with a CIN III/HSIL lesion. As can be seen, 5 weeks after phototheranostics, there are no signs of a cervical intraepithelial squamous lesion.

In 35 (78.7%) women, according to the cytological examination, they had a normal cytological picture of NILM 6 weeks after the first course of phototheranostics, and the remaining 10 (21.3%) cases had a mild squamous intraepithelial lesion of the cervix—CIN I/LSIL. Of the six patients who underwent a second course of phototheranostics after 6 weeks, according to the cytological conclusion, NILM was observed in four (66.7%) cases and CINI/LSIL in two (33.3%) cases. After 3 months, 2 patients (4.4%) had cytologically verified LSIL, and the remaining 43 (95.2%) women had NILM. In all patients, the normal cytological picture of NILM was determined cytologically after 6, 9, and 12 months.

In all patients, HPV elimination was recorded according to the DNA-PCR amplification method of the cervical canal detachable. In all patients after 3, 6, 9, and 12 months, HPV was also not detected.

An analysis of the results of a colposcopy after PDT showed that the number of patients with abnormal signs in the CIN III/HSIL group was two (11.7%) cases, and among patients with CIS, one (3.6%) (Table 5). Thus, the overall incidence of abnormal colposcopic signs in the entire sample was 6.6% (3 cases). After 3 months, three patients (6.6%) had mild cervix lesions in the form of delicate mosaic, punctures, and thin acetowhite epithelium. After 6 months, two patients (4.4%) were found to have mild cervix lesions in the form of a thin acetowhite epithelium. After 9 and 12 months, all patients were diagnosed with a normal colposcopic picture.

According to the extended colposcopy performed before PDT, during a test with a 3.5% solution of acetic acid, abnormal signs were observed in the form of a dense aceto-white epithelium, rough puncture, and rough mosaic in 15 out of 17 patients with subsequent histologically confirmed CIN III\HSIL neoplasm and in 27 out of 28 patients with CIS, as well as iodine-negative zones in the Schiller test. In two patients with CIN III\HSIL and in one patient with CIS, mild signs of cervical lesions were noted in the form of delicate mosaics, punctures, and thin acetowhite epithelium.

The results of the colposcopy performed after the first course of PDT showed that 15 out of 17 patients with CIN III\HSIL and 27 out of 28 patients with CIS had normal colposcopic pictures with the absence of acetowhite epithelium in a sample with a 3.5% solution acetic acid and iodine-negative zones in the Schiller test. In two patients with CIN III\HSIL and in one patient with CIS after the first PDT procedure, mild signs of cervical lesions were revealed in the form of delicate mosaics, punctures, and thin acetowhite epithelium.

After 3 months, three patients (6.6%) had mild cervix lesions in the form of delicate mosaics and thin acetowhite epithelium. After 6 months, two patients (4.4%) were found to have mild cervix lesions in the form of a thin acetowhite epithelium. After 9 and 12 months, all patients were diagnosed with a normal colposcopic picture.

**Figure 4:**
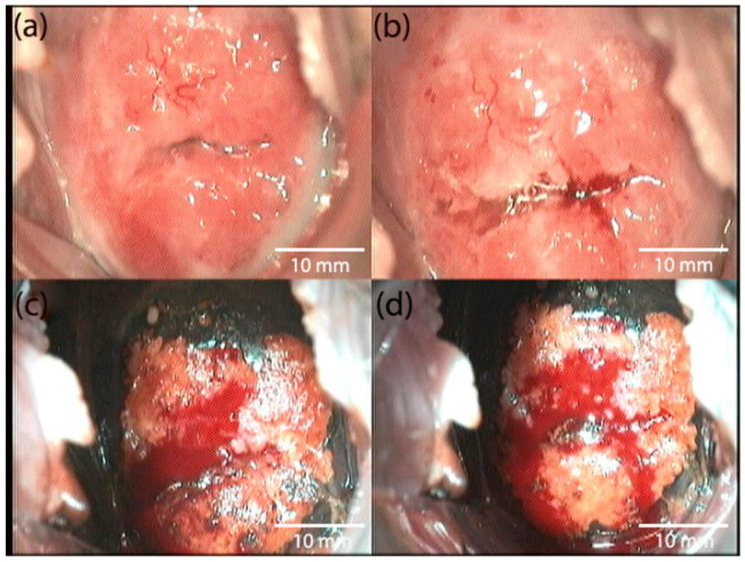
Abnormal colposcopic imaging from a patient with preinvasive cancer before PDT: (**a**) native image, (**b**) image when the cervix is treated with a solution of 3.5% acetic acid, and (**c**,**d**) images when the cervix is treated with Lugol’s solution—Schiller’s test.

**Figure 5:**
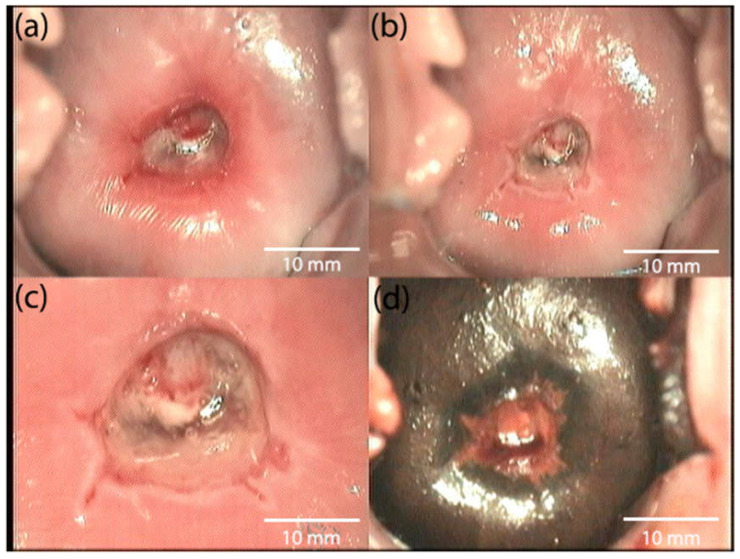
Normal colposcopic imaging in a patient with preinvasive cancer 4 weeks after PDT: (**a**) native image, (**b**) image when the cervix is treated with a solution of 3.5% acetic acid, and (**c**,**d**) images when the cervix is treated with Lugol’s solution—Schiller’s test.

Figure 4 shows the patient’s colposcopic imaging before PDT. Figure 5 shows the patient’s colposcopic imaging 4 weeks after treatment.

**Figure 6:**
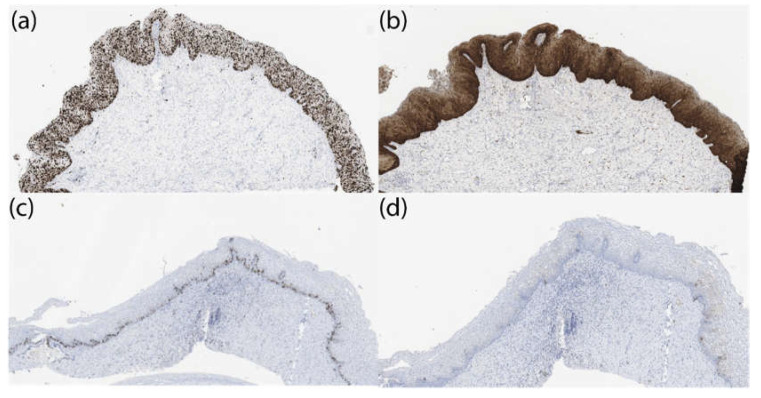
Immunohistochemical analysis: (**a**) expression of Ki 67 at the level of 85% in a patient with CIN III/HSIL before phototheranostics, (**b**) expression of Ki 67 at the level of 35% in a patient with CIN III/HSIL 5 weeks after phototheranostics, (**c**) expression of p16 in a patient with CIN III/HSIL before phototheranostics, and (**d**) expression of p16 in a patient with CIN III/HSIL 5 weeks after phototheranostics. Boost 90.

The results of the immunohistochemical analysis are presented in Figure 6 and in Table 6 and Table 7.

The study of the dynamics of Ki-67 expression showed high levels of this marker in both groups before treatment; the median values were 84.0 (83.0; 87.0) and 85.0 (85.0) (82.0; 88.0)% (Table 6). After PDT, the levels of Ki-67 expression decreased statistically significantly (*p* < 0.05) relative to the initial values, amounting to 30.0 (27; 33.0)% in women with severe intraepithelial squamous cell lesions; in the group of patients with CIS, the value of the indicator was 29.0 (26; 31.0)%.

An assessment of the dynamics of p16 expression also indicated its sharp decrease after the treatment with PDT. Before treatment, the values of this indicator were 97.0 (95.0; 99.0) and 96.0 (95.0; 98.0)% in the CIN III/HSIL and CIS groups, respectively, while, after PDT, the p16 expression was not determined (Table 7).

In general, the obtained results indicated the high efficiency of PDT with intravenous administration of the chlorin PS for the treatment of intraepithelial lesions of the cervix with a selective effect on the pathologically altered tissue.

## 4. Discussion

Since CC dominates in the structure of malignant tumors in women aged 15–39 years, it is important to improve the treatment methods of precancerous cervical pathologies and early invasive CC [2,3,4,5]. This tumor is one of the most successfully treatable forms of cancer if the disease is detected at an early stage. Therefore, one of the directions of scientific research is the development and testing of treatment methods for HPV-associated cervical squamous intraepithelial lesions and CIS, the use of which does not impair fertility and ensures the preservation of the reproductive health of this group of patients.

Phototheranostics is an alternative organ-preserving therapeutic approach to cancer treatment. The method is based on a combination of selective PS accumulation in pathological foci and the specific damaging effect of laser radiation on malignant cells or foci of precancerous tissue. This method provides the possibility of its repeated application [7,8,9,10,11,12].

The results obtained in the course of the present work indicate the high efficiency of phototheranostics with intravenous administration of chlorin PS in the treatment of intraepithelial cervical lesions and CIS. It has been established that, in 85–88% of patients, a complete effect is observed already after the first session of phototheranostics; in 88–89% of cases, a complete regression of lesions on the cervical mucosa is achieved.

Our data are consistent with the results of other authors. Thus, systematic reviews performed to date show that the rate of complete remission during PDT for CIN and cervical HPV infection is 81.0% (from 31.3 to 100%) and 80.4% (range 53.4–94.4%), respectively [18,21]. One of these works provides data on the frequency of complete remission of CIN in up to 100% of cases, while HPV eradication was observed in 53.4–80.0% of cases [27].

In a study performed by Su Y. et al. (2021), the clinical efficacy and safety of PDT with 5-ALA in cervical intraepithelial neoplasia and vaginal intraepithelial neoplasia was evaluated. A retrospective analysis of 48 patients diagnosed with CIN and vaginal intraepithelial neoplasia who underwent PDT with 5-ALA was performed. It was found that the rate of complete remission in patients after treatment was 88.64% (39 out of 44) 3–6 months after treatment, while the overall HPV clearance rate was 46.34 and 60.98% after 3 and 12 months of follow-up, respectively. It is noteworthy that atypical lesions of the vessels and the endocervical canal significantly affected the effectiveness of PDT with 5-ALA. In addition, five patients had residual lesions (11.36%) during follow-up, and one patient had a relapse (2.56%) [16].

Currently, PDT is mainly used to treat patients with CIN I–II. The limited use of the method in severe cervix dysplasia is due, in most cases, to fears of malignancy [28,29,30,31]. However, some authors who have performed PDT with systemic PS use report a high rate of complete treatment effect. Therefore, in the study performed by Grebenkiya E.V. et al. (2014), the experience of treating CIN and early CC by PDT in 12 patients diagnosed with CIN II-III and cancer in situ was described. The chlorin-type PS photolon was administered intravenously at a dose of 0.75–1.15 mg/kg of the patient’s body weight. After 1.5–2 h, an irradiation session was performed using a polypositional laser exposure technique (energy density of laser radiation at one position: 150 J/cm^2^ and power density 400–500 mW/cm^2^). Thirty days after treatment, conization of the cervix with curettage of the cervical canal was performed, evaluating the results of the PDT. According to the results of the histological examination of the postoperative material, the effect of the treatment in four patients was assessed as complete regression, seven patients had small foci of CIN I, and one patient had a foci of CIN II. In 8 out of 10 HPV-positive patients, complete eradication of HPV was obtained after the treatment. No serious adverse events were reported during the irradiation procedure.

The authors concluded that the pronounced therapeutic effect, high antiviral activity, and good tolerability make it possible to consider PDT an alternative organ-preserving treatment for early CC and CIN [31].

Filonenko E.S. et al. (2015) presented the results of a clinical study on the effectiveness of PDT with radachlorin in patients with precancerous and neoplastic cervical pathology. The study enrolled 30 patients, including 4 patients with cervical erosion, 5 patients with stage II dysplasia, 13 patients with stage III dysplasia, 4 patients with carcinoma in situ, and 4 patients with a diagnosis of CC stage Ia. Radachlorin was administered once intravenously as a 30-min infusion at a dose of 1.0 mg/kg body weight 3 h before irradiation (wavelength 662 nm, energy density 300–350 J/cm^2^). The result of the treatment in 26 (86.7%) patients was qualified as a complete regression of the tumor and in 4 (13.3%) as a partial regression. In groups with a clinical diagnosis of cervical erosion, stage II dysplasia and carcinoma in situ complete regression was observed in all cases. In the group with stage III dysplasia, complete regression after the first course of PDT was achieved in 77% of patients and, in women with CC stage Ia, in 75% of patients. No adverse reactions associated with the use of radachlorin or PDT were recorded during treatment and follow-up [32].

Thus, our data, as well as literature data, confirm that the high selectivity of the effect on the affected tissues of the cervix, the low risk of developing serious adverse reactions and complications, the short period of systemic photosensitivity, and high therapeutic efficacy favorably distinguish PDT with Ce6 from the traditional methods of treatment CIN III/HSIL and CIS, which are based on the local destruction of affected tissues. PDT using Ce6 is an effective, relatively safe, and minimally invasive method of treatment.

## 5. Conclusions

The results of the study indicate that the phototheranostic method is a clinically effective and safe integrated approach to the diagnosis and treatment of CIN and CIS. The performance of spectral and video fluorescent diagnostics and simultaneous PDT contribute to the successful treatment of neoplasia foci on the cervical mucosa and the elimination of the etiological factor—HPV. In this case, a selective effect is proceeded on pathologically altered tissue sections of this area, which does not cause damage to the normal surrounding tissues, coarse scarring, and stenosis of the cervical canal. Thus, the use of PDT makes it possible to preserve the normal anatomical and functional characteristics of the cervix, which ensures the preservation of fertility in the considered group of patients. It also confirms a number of advantages of the diagnostic and treatment approach used in the present work for severe intraepithelial cervical neoplasia and CC compared to alternative methods of treatment such as ablation, excision, and conization of the cervix.

## Figures and Tables

**Table 1:** Distribution of patients according to the characteristics of their cervical lesions.

Patient Group	Number of Patients *n* (%)
CIN III/HSIL	17 (37.8)
CIS	28 (62.2)

**Table 2:** Frequency of detection of the oncogenic HPV types.

HPV Type	Number of Patients *n* (%)
16 18 33 11 35 56	18 (40.0) 14 (31.1) 6 (13.4) 2 (4.4) 3 (6.7) 2 (4.4)

**Table 3:** Distribution of patients according to the number of PDT procedures; after which, the treatment effect was observed.

Patient Group (*n*)	The Number of Patients in Whom the Effect Was Observed after One PDT Procedure *n* (%)	The Number of Patients in Whom the Effect Was Observed after Two PDT Procedures *n* (%)
CIN III/HSIL (17)	15 (88.2)	2 (11.8)
CIS (28)	24 (85.7)	4 (14.4)
Total (45)	39 (86.7)	6 (13.3)

**Table 4:** Treatment outcomes.

Patient Group (*n*)	Complete Regression *n* (%)	Partial Regression *n* (%)
CIN III/HSIL (17)	15 (88.2)	2 (11.8)
CIS (28)	25 (89.3)	3 (10.7)
Total (45)	40 (88.9)	5 (11.1)

**Table 5:** The frequency of detection of abnormal signs during colposcopy after PDT.

Patient Group (*n*)	Abnormal Signs *n* (%)
CIN III/HSIL (17)	2 (11.7)
CIS (28) Total (45)	1 (3.6) 3 (6.6)

**Table 6:** Dynamics of the Ki-67 expression level in patients who underwent PDT.

Patient Group (*n*)	Before PDT % Me (Q_25_; Q_75_)	After PDT % Me (Q25; Q75)
CIN III/HSIL (17)	84.0% (83.0; 87.0)	30.0% (27; 33.0) *
CIS (28)	85.0% (82.0; 88.0)	29.0% (26; 31.0) *

* *p* < 0.05.

**Table 7:** Dynamics of the p16 expression level in patients who underwent PDT.

Patient Group (*n*)	Before PDT % Me (Q_25_; Q_75_)	After PDT % Me (Q25; Q75)
CIN III/HSIL (17)	97.0% (95.0; 99.0)	0%(0; 0) *
CIS (28)	96.0%(95.0; 98.0)	0%(0; 0) *

* *p* < 0.05.

## Data Availability

The data presented in this study are available on request from the corresponding author.
